# Probe capture-based tNGS for microbial detection of dacryocystitis

**DOI:** 10.1128/spectrum.02132-26

**Published:** 2026-09-21

**Authors:** Xinyue Yu, Jiayi Zhang, Kerui Wang, Yunming Liu, Xinyu Zheng, Can Yang, Weifeng Huang, Jun Liu, Feifei Liu, Rongxin Chen, Xuanwei Liang, Xinran Dong

**Affiliations:** 1 State Key Laboratory of Ophthalmology, Zhongshan Ophthalmic Center, Sun Yat-sen University, Guangdong Provincial Key Laboratory of Ophthalmology and Visual Science 567962 , Guangzhou, China; 2 Guangzhou Kingmed Center for Clinical Laboratory 633507 , Guangzhou, China; 3 Guangdong Eye Institute, Department of Ophthalmology, Guangdong Provincial People’s Hospital (Guangdong Academy of Medical Sciences), Southern Medical University 70570 , Guangzhou, China; Children's National Hospital, George Washington University, Washington, DC, USA

**Keywords:** dacryocystitis, targeted next-generation sequencing, bacterial cultures, microbial landscape

## Abstract

Dacryocystitis is one of the common infectious diseases in ophthalmology. This study evaluates microbial distribution in lacrimal sac secretions using targeted next-generation sequencing (tNGS) and compares it with traditional culture methods. Conducted at the Zhongshan Ophthalmic Center, 31 patients with confirmed dacryocystitis were enrolled. Lacrimal sac secretions were collected during endoscopic dacryocystorhinostomy and analyzed using both tNGS and conventional bacterial cultures. Microbial diversity was assessed, and antibiotic susceptibility was evaluated for cultured isolates. We compared the positivity rate and the detection of various microorganisms between the two tests, as well as the associated resistance information. tNGS detected a significantly broader range of microorganisms (*P* = 0.0002). Beyond bacteria and fungi, tNGS revealed substantial viral and parasitic presence: viruses were detected in 22 of 31 patients, with Epstein-Barr virus and human herpesvirus 7 being the most prevalent, while parasitic sequences were identified in six patients, including *Acanthamoeba* species in four patients. Resistance genes were identified in three patients, suggesting the potential presence of antimicrobial resistance determinants. tNGS provides broader microbial profiling in dacryocystitis and may complement conventional culture by detecting not only bacteria but also fungi, viruses, parasites, and resistance genes. These findings highlight the necessity of managing ocular surface microbiota imbalances across a broad spectrum of microorganisms and emphasize the importance of judicious antibiotic use in clinical practice. Additionally, these observations raise the hypothesis that the lacrimal sac may serve as a potential reservoir for fungi, *Acanthamoeba*, and viruses on the ocular surface.

**IMPORTANCE:**

Dacryocystitis is one of the most common lacrimal duct obstruction disorders in ophthalmology. Conventional culture detected bacteria in only 38.7% of patients in this study and is unable to detect non-culture-dependent pathogens such as viruses and parasites or provide resistance information. As a critical complement to traditional culture, targeted next-generation sequencing (tNGS) detected microorganisms in 87.1% of cases, covering bacteria, fungi, viruses, and parasites while simultaneously identifying resistance genes—substantially enhancing both detection breadth and depth. Notably, tNGS revealed for the first time substantial levels of viruses, fungi, and *Acanthamoeba*—a dangerous organism linked to severe corneal infections—in the lacrimal sac, raising the novel hypothesis that the lacrimal sac may serve as an unrecognized reservoir for diverse ocular surface microbes. These findings expand the traditional bacteria-centered view of dacryocystitis and highlight the potential value of tNGS in improving diagnostic evaluation, supporting antibiotic stewardship, and guiding future studies of persistent or recurrent ocular infections.

## INTRODUCTION

Dacryocystitis is one of the most common lacrimal duct obstruction disorders in ophthalmology, typically secondary to nasolacrimal duct obstruction (NLDO) caused by various factors(1, 2). Tear retention due to obstruction creates an ideal environment for microbial growth in the lacrimal sac and ocular surface. During acute inflammation, these pathogens can lead to mild manifestations such as epiphora and increased ocular discharge. In more severe cases, they can result in skin infections around the lacrimal region, orbital cellulitis, or intracranial infections (3). Pathogens on the ocular surface may enter through corneal wounds or incisions from intraocular surgery, potentially causing serious complications like corneal ulcers or endophthalmitis. On the other hand, the development of dacryocystitis is closely associated with dysbiosis of the ocular surface and lacrimal microbiome, or with viral infections of the ocular surface, which can seriously affect patients’ quality of life and work (4). Although dacryocystorhinostomy (DCR) is highly effective in treating dacryocystitis (5), managing ocular surface microbiota imbalances and the prudent use of antibiotics remain crucial aspects of patient care.

In recent years, culture-independent techniques such as polymerase chain reaction (PCR) and metagenomic next-generation sequencing (mNGS) have been increasingly utilized for the etiological diagnosis of various infectious diseases (6). Traditional microbial diagnostics involve the cultivation and isolation of microorganisms in cultures, detection of pathogen-specific antibodies (serology) or antigens, and molecular identification of microbial nucleic acids (DNA or RNA) (7, 8). Since 2005, the advent of mNGS has enabled comprehensive characterization of all DNA or RNA present in a sample. This allows for a holistic analysis of the microbiome as well as the human host genome or transcriptome in patient samples, offering advantages such as shorter detection times, reduced bias, and broader coverage (9). However, mNGS testing is relatively expensive. Additionally, the high proportion of human nucleic acids in clinical samples can reduce the sensitivity of pathogen detection, leading to the potential risk of missing antimicrobial resistance (AMR) or virulence genes (10). Target enrichment strategies are commonly used in NGS workflows to eliminate genomic DNA regions that are not of interest. As an emerging genomic detection method, probe capture-based targeted next-generation sequencing (tNGS) significantly reduces sequencing data volume while increasing pathogen information and sensitivity within samples, optimizing both performance and cost (11).

In this study, we applied the tNGS method to assess microbial distribution in lacrimal sac secretions of patients with dacryocystitis and compared it with traditional in-hospital bacterial culture methods, aiming to characterize the full spectrum of microorganisms in the lacrimal sac. In the context of dacryocystitis, conventional cultures are often negative or identify only a single organism, which limits the clinician’s ability to select appropriate antimicrobial therapy. By enabling rapid and comprehensive detection of bacteria, fungi, viruses, and parasites—along with resistance genes—tNGS may provide supplementary microbiological information to support more precise treatment of dacryocystitis. Early and precise identification of clinically relevant microorganisms in dacryocystitis is important, as delayed or inadequate treatment can lead to severe complications, including orbital cellulitis, cavernous sinus thrombosis, and life-threatening intracranial infections.

## MATERIALS AND METHODS

### Study design

This study was conducted at the Zhongshan Ophthalmic Center of Sun Yat-sen University, China, from 5 July to 10 August 2024. A total of 31 patients with nasolacrimal duct obstruction were consecutively enrolled, including 3 cases of congenital infantile dacryocystitis, 3 cases of acute dacryocystitis, and 25 cases of chronic dacryocystitis. Inclusion criteria included (i) confirmed diagnosis of nasolacrimal duct obstruction through symptoms, signs, and lacrimal duct irrigation tests, and (ii) planned treatment via endoscopic dacryocystorhinostomy (En-DCR). Exclusion criteria were (i) concurrent canalicular or punctal obstruction; (ii) presence of other conditions affecting ocular surface homeostasis or severe systemic diseases such as Sjögren’s syndrome or Stevens-Johnson syndrome; and (iii) severe psychiatric disorders or inability to cooperate with this study. Lacrimal sac secretions were obtained from all patients during surgery. All En-DCR surgeries were performed under general anesthesia by the same senior surgeon (X.L.). During the procedure, after standard nasal disinfection and exposure of the lacrimal sac, the lacrimal sac was incised under endoscopic visualization. Before lacrimal irrigation or contact with nasal secretions, gentle pressure was applied over the lacrimal sac region, and the expressed lacrimal sac secretion was immediately aspirated using a sterile 10 mL disposable syringe. During collection, the syringe tip was kept within the lacrimal sac opening and was carefully prevented from touching the nasal mucosa, surgical instruments contaminated by nasal secretions, or surrounding skin. The collected samples were evenly divided into two portions, each placed in a sterile disposable EP tube, and stored at −80°C. This study was approved by the Institutional Review Board of the Zhongshan Ophthalmic Center, Sun Yat-sen University, and was conducted in compliance with the tenets of the Declaration of Helsinki. Written informed consent was obtained from all participants prior to their inclusion in the study.

The patients’ general information, medical history, symptoms, clinical signs, surgical plans, and prior antibiotic usage were thoroughly reviewed and meticulously documented.

### Bacterial culture and antibiotic susceptibility testing

Collected lacrimal sac retention fluid samples were inoculated onto 5% sheep blood agar, chocolate agar, and MacConkey agar (Becton Dickinson, Franklin Lakes, NJ, USA) for aerobic bacterial culture, and Brucella blood agar supplemented with hemin (5 µg/mL) and vitamin K¹ (1 µg/mL; Anaerobe Systems, Morgan Hill, CA, USA) for anaerobic culture. Sabouraud dextrose agar was used for fungal culture. Aerobic and fungal plates were incubated at 35°C in a 5% CO_2_ incubator; anaerobic plates were incubated at 35°C in an anaerobic jar with GasPak EZ anaerobe container system sachets (Becton Dickinson). All plates were examined daily for 7 days; fungal cultures were held for up to 14 days if no growth was observed initially. For isolates with scant growth at 7 days, repeat subculture to fresh blood agar and chocolate agar was performed, with extended incubation for up to 10 days total.

Uninoculated control plates for each medium type were included to confirm the sterility of the testing environment. Bacterial identification was performed using the VITEK MS matrix-assisted laser desorption/ionization time-of-flight mass spectrometry system with MYLA software version 3.0 (bioMérieux, Marcy-l'Étoile, France). Antibiotic susceptibility testing was conducted by the Kirby-Bauer disk diffusion method according to the Clinical and Laboratory Standards Institute (CLSI) M100 30th edition (2020) guidelines. Quality control was performed in parallel with each antimicrobial susceptibility testing (AST) batch using *Staphylococcus aureus* ATCC 25923, *Escherichia coli* ATCC 25922, and *Pseudomonas aeruginosa* ATCC 27853 for aerobic bacteria, and *Bacteroides fragilis* ATCC 25285 for anaerobic culture. Zone diameters were interpreted using CLSI breakpoints. Multidrug-resistant bacteria were defined as those resistant to three or more classes of antibiotics currently in clinical use.

For isolates with insufficient growth for standard antimicrobial susceptibility testing, repeat subculture was performed by transferring visible colonies to fresh 5% sheep blood agar and chocolate agar plates, followed by additional incubation for 48–72 h at 35°C in 5% CO_2_. When growth remained insufficient to achieve the inoculum required for routine AST, the primary plates were further incubated for up to 10 days with periodic examination. Despite these attempts, three *Stenotrophomonas maltophilia* isolates still did not yield enough growth for standard disk diffusion testing, and no susceptibility results were reported for these isolates.

### tNGS workflow

A 600 µL lacrimal sac retention fluid sample and 250 µL of 0.5 mm glass beads were attached to a horizontal platform on a vortex mixer and agitated vigorously at 4,000 rpm for 2 min. The glass beads were washed with DNase/RNase-free water, treated with UV irradiation for 30 min, and autoclaved at 121°C for 20 min to eliminate any microbial DNA contamination prior to use. A 0.3 mL aliquot was transferred into a new 1.5 mL microcentrifuge tube, and nucleic acid was extracted using the MataPure DNA and RNA Extraction Kit (KingCreate, China).

Following cDNA conversion from RNA, libraries were constructed through fragmentation, end-repair, adapter-ligation, and PCR amplification. Eight uniquely barcoded libraries were pooled to hybridize and specific biotinylated probes for 2 h after library generation using the MetaCAP Pathogen Capture Metagenomic Assay Kit (KingCreate, China). Agilent 2100 was used for quality control, confirming library peaks around 350 bp. Quality-validated libraries were pooled using Qubit dsDNA HS Assay Kit (Thermo Fisher Scientific Inc.), and sequencing was performed on the Illumina Miniseq platform set to 100-bp single-end with an average of 1 million reads per sample.

PDseq (KingCreate, China) is a commercially available automated bioinformatics analysis platform designed for the detection of pathogenic microorganisms from sequencing data. The primary workflow includes data quality control, host filtering, pathogenic alignment, reporting, and visualization. Clean reads were obtained by removing sequencing adapters, low-quality reads, those with excessive N bases, and those below 35 bp in length. Remaining reads were aligned to the human reference genome (hg38) to filter human-origin sequences. Subsequently, the remaining reads were compared to a classification reference genome database containing 13,214 bacteria, 9,811 viruses, 3,180 fungi, and 405 parasites, which were downloaded from NCBI (ftp://ftp.ncbi.nlm.nih.gov/genomes/). Custom software was employed to calculate reads per million (RPM) at the species or genus level, and sequence alignment was visualized.

### Statistical analysis

Normality of continuous variables was assessed using the Shapiro–Wilk test. Normally distributed data are presented as mean ± standard deviation with range; non-normally distributed data are presented as median with interquartile range (IQR, 25th–75th percentiles). Categorical variables are reported as counts (*n*) and percentages (%) with 95% confidence intervals (CI) calculated by the Clopper–Pearson exact method. Comparisons of positive detection rates between tNGS and culture were performed using Fisher’s exact test (two-tailed) for 2 × 2 tables; Pearson’s *χ*^2^ test was used where all expected cell frequencies were ≥5. Effect sizes are reported as odds ratios (OR) with 95% CI. No adjustment for multiple comparisons was performed given the exploratory nature of this pilot study. A two-sided *P*-value < 0.05 was considered statistically significant. All analyses were conducted using SPSS version 25.0 (IBM Corp., Armonk, NY, USA) and R version 4.3.1 (R Foundation for Statistical Computing, Vienna, Austria).

## RESULTS

### Characteristics of the study participants

As shown in Table 1, this study included 31 patients with dacryocystitis: 3 with congenital dacryocystitis, 3 with acute dacryocystitis, and 25 with chronic dacryocystitis.

**TABLE 1:** Clinical demographics of the patients with dacryocystitis^*a*^

Variable	(*n*, %)
Eyes and laterality	
Right	11 (35.5)
Left	17 (54.8)
Both	3 (9.7)
Sex	
Male	5 (16.1)
Female	26 (83.9)
Age (years)	50.45 ± 17.39
Duration of symptoms (months)	67.03 ± 77.60
Types of dacryocystitis	
Acute dacryocystitis	3 (9.7)
Chronic dacryocystitis	25 (80.6)
Congenital dacryocystitis	3 (9.7)
Properties of secretions	
Epiphora	25 (80.6)
Ocular secretions	29 (93.5)
Swelling pain	1 (3.2)
Previous treatment	
Lacrimal Laser	2 (6.4)
Dacryocystorhinostomy	1 (3.2)
Skin incision for pus drainage	2 (6.4)
N/A	26 (83.9)
Secretory property	
Pus-filled discharge	28 (90.3)
Clear secretion	3 (9.6)
Surgical technique	
En-DCR	5 (16.2)
En-DCR and intubation stent	26 (83.8)
Antibiotherapy methods	
Oral antibiotic	6 (19.4)
IV antibiotics	1 (3.2)
Antibiotic eye drop	18 (58.0)
N/A	6 (19.4)
Antibiotherapy time (years), *n* = 25	
Less than 1 year	5 (20)
1–3 years	3 (12)
Irregularly	2 (8)
Details not available	15 (60)
Positive bacterial samples	
Bacterial culture	12 (38.7)
tNGS	27 (87.1)

^*a*^ Targeted next-generation sequencing (tNGS).

The cohort consisted of 5 male (16.1%) and 26 female patients (83.9%), with a mean age of 50.45 ± 17.39 years. The mean disease duration was 67.03 ± 77.60 months (range: 0.5–360 months). The wide range and large standard deviation reflect the etiological heterogeneity of the cohort, which included acute cases with short symptom duration, chronic cases with prolonged disease courses, and congenital cases present since birth. Eleven patients had involvement of the right eye, 17 had involvement of the left eye, and 3 had bilateral involvement. For patients with bilateral dacryocystitis, the right eye was selected for sampling to maintain consistency across the study.

Tearing was reported by 25 patients (80.9%), increased eye discharge by 29 patients (93.5%), and one patient (3.2%) reported a distinct sensation. Among the 31 patients, 5 (16.1%) had previously undergone lacrimal duct recanalization surgery, while 26 (83.9%) had no prior surgical history. Purulent discharge was observed in 28 patients (90.3%), while 3 (9.6%) had clear discharge. Regarding surgical intervention, 5 patients (16.2%) underwent only En-DCR, while 26 patients (83.8%) had En-DCR with lacrimal duct silicone tube implantation.

The prior use of antibiotics was recorded as follows: 6 patients (19.4%) had not used antibiotics, 6 (19.4%) had taken oral antibiotics, 1 patient (3.2%) had received intravenous antibiotics, and 18 patients (58.0%) had used antibiotic eye drops. Among those who had used antibiotics, 5 patients had taken them for less than 1 year, 3 patients for 1–3 years, 2 patients used them irregularly, and 15 patients were unsure about the specifics. Of those using antibiotic eye drops, two reported using levofloxacin eye drops, one used gatifloxacin eye drops, while others were unclear on the specific medications used. The substantial proportion of patients with prior antibiotic exposure (80.6%) represents an important consideration, as antecedent antimicrobial therapy may have altered the microbial composition of lacrimal sac secretions and reduced the sensitivity of bacterial culture.

### Microbiologic culture results of lacrimal sac secretions

Conventional bacterial culture was positive in 12 of 31 patients (38.7%; 95% CI: 22.4%–57.5%; Table 2). The most frequently isolated bacterium was *Staphylococcus epidermidis* (4/12, 33.3%; 95% CI: 9.9%–65.1%), followed by *Stenotrophomonas maltophilia* (3/12, 25.0%; 95% CI: 5.5%–57.2%) and *Streptococcus pneumoniae* (2/12, 16.7%; 95% CI: 2.1%–48.4%). *Staphylococcus epidermidis* was the most frequently identified bacterium, found in four samples. This strain showed resistance to erythromycin, clindamycin, moxifloxacin, levofloxacin, oxacillin, and penicillin G. Three samples tested positive for *Stenotrophomonas maltophilia*, although antimicrobial susceptibility testing was not performed for these isolates due to insufficient bacterial growth for standard AST panels. Two patients were positive for *Streptococcus pneumoniae*, which exhibited resistance to tetracycline, erythromycin, clarithromycin, azithromycin, and trimethoprim/sulfamethoxazole, thereby meeting the criteria for multidrug-resistant bacteria (MDRB). *Pseudomonas alcaligenes*, *Propionibacterium avidum*, and *Klebsiella pneumoniae* were each identified in one patient. One patient had methicillin-resistant *Staphylococcus aureus* (MRSA), and one culture showed. only a few colonies that were insufficient for species identification. Fungal infection was detected in one patient, who tested positive for *Candida guilliermondii*.

**TABLE 2:** The bacteria from the lacrimal sac secretions in bacterial culture^*a*^

Types of isolated bacteria	Number of positive samples (*n* = 12)	Types of resistant antibiotics
*Streptococcus pneumoniae*	2	Tetracycline, erythromycin, clarithromycin, azithromycin, and trimethoprim/sulfamethoxazole
*Staphylococcus epidermidis*	4	Erythromycin, clindamycin, moxifloxacin, levofloxacin, oxacillin, and penicillin G
*Stenotrophomonas maltophilia*	3	
*Pseudomonas alcaligenes*	1	
*Propionibacterium avidum*	1	
*Klebsiella pneumoniae*	1	
Few stray bacteria growth	1	
MDRB	1	
MRSA	1	

^*a*^ Multidrug-resistant bacteria (MDRB); Methicillin-resistant *Staphylococcus aureus* (MRSA).

### Microbial tNGS results of lacrimal sac secretions

Using the tNGS method, a total of 32 bacterial species, 25 fungal species, 34 viruses, and 9 parasites were identified across the 31 patient samples. Figure 1 illustrates the species and relative distribution of all microorganisms detected in each patient. These detections represent a composite of probable pathogens, commensal colonizers, and potential contaminants, with classification based on clinical correlation, established pathogenicity, and relative abundance (see Discussion for detailed criteria). Bacterial species were detected by tNGS in 27 of 31 patients (87.1%; 95% CI: 70.2%–95.1%), significantly higher than the 12 of 31 (38.7%; 95% CI: 22.4%–57.5%) detected by conventional culture (Fisher’s exact test, *P* = 0.0002; OR 12.6, 95%: CI 2.8–89.4; Table S1). As illustrated in Fig. 2, the most frequently detected bacterium by tNGS was *Streptococcus pneumoniae*, found in six patient samples and classified as a probable pathogen given its established role in dacryocystitis and its dominance in four of these samples. *Cutibacterium acnes* and *Staphylococcus aureus* were each detected in four samples; while *S. aureus* was flagged as a likely pathogen based on culture concordance and clinical correlation in two patients, *C. acnes* was considered probable commensal colonization given its known presence in the ocular surface microbiome and absence of dominant abundance.

**Fig 1:**
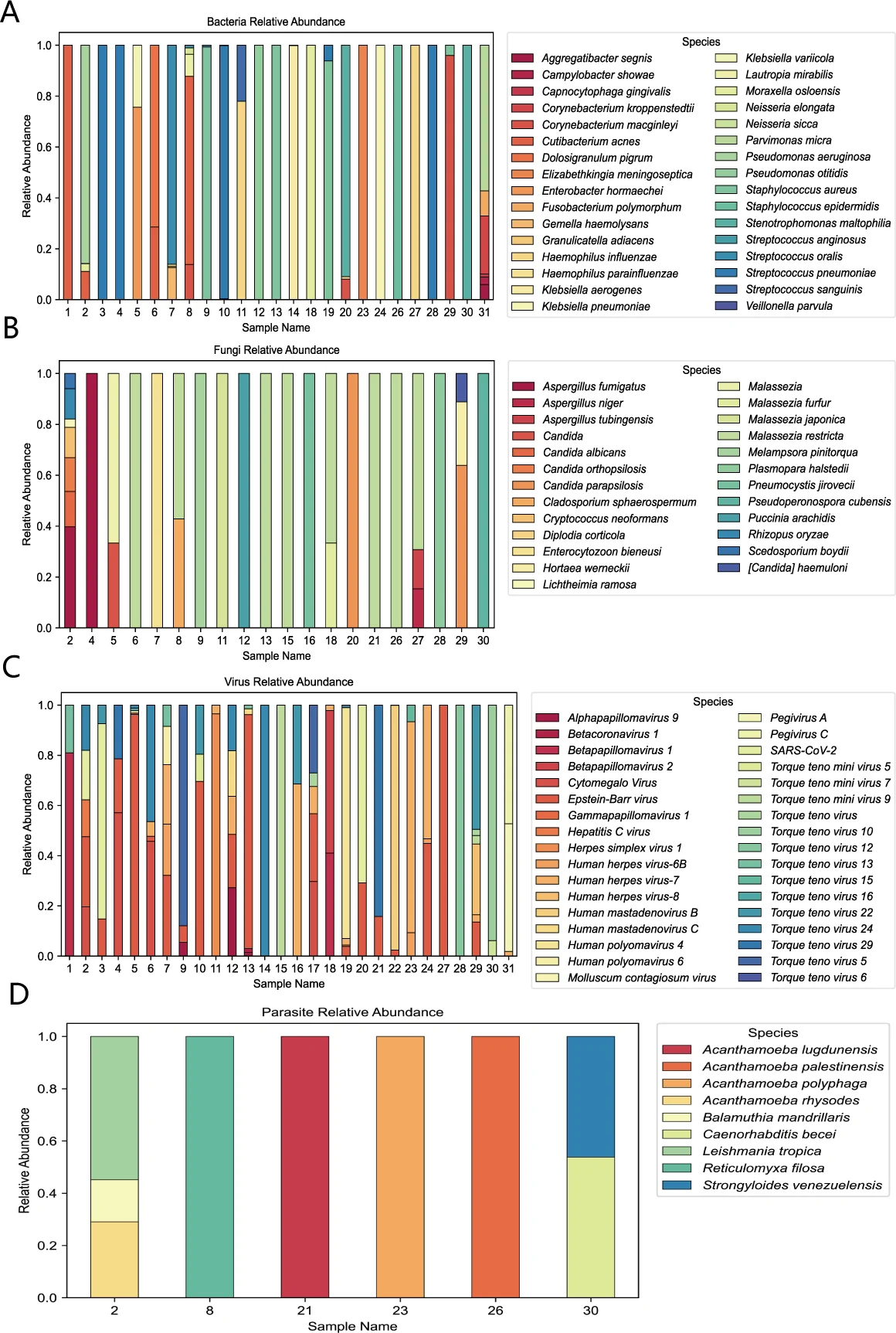
The relative abundance of various microbial communities detected by tNGS in lacrimal sac secretions of 31 patients with dacryocystitis. (**A**) The relative abundance of various bacteria detected in lacrimal sac secretion samples. (**B**) The relative abundance of various fungi detected in lacrimal sac secretions. (**C**) The relative abundance of various viruses detected in lacrimal sac secretion samples. (**D**) The relative abundance of various parasites detected in lacrimal sac secretion samples.

**Fig 2:**
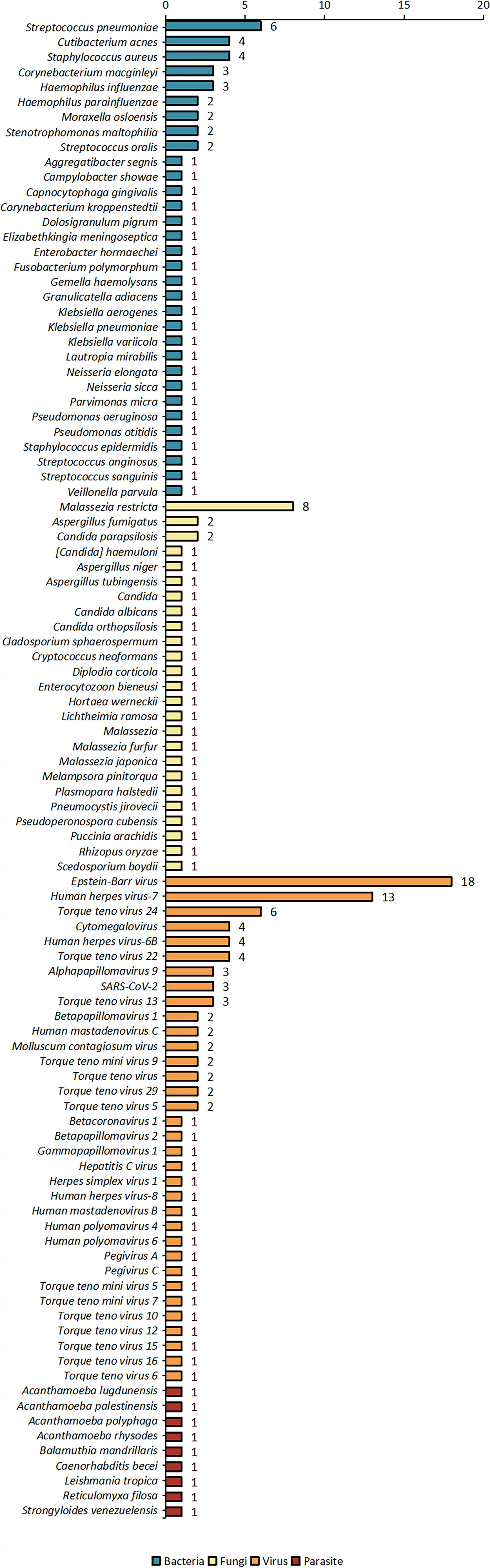
The names and relative distribution of all types of microorganisms detected using tNGS technology in lacrimal sac secretion samples from 31 patients with dacryocystitis.

Fungal species were identified in 20 patient samples, with *Malassezia restricta* being the most common (eight positive samples, 25.8%), potentially representing commensal colonization or anatomical extension from the adjacent sinus microbiome rather than active fungal infection. *Candida parapsilosis* was detected in two patients; given its known pathogenic potential in ocular infections, these detections were classified as possible pathogens pending clinical correlation.

Beyond bacteria and fungi, tNGS revealed a previously underappreciated presence of viruses and parasites in the lacrimal sac. The most common virus detected was Epstein-Barr virus (EBV), found in 18 patients (58.1%), followed by human herpesvirus-7 (HHV-7; 13 patients, 41.9%) and Torque teno virus 24 (6 patients, 19.4%). These viral detections were interpreted as likely latent or subclinical carriage given the absence of acute viral signs and the established biology of these herpesviruses, though their potential contribution to chronic inflammation or mucosal dysfunction is discussed below.

Parasites were identified in six patients (19.4%). *Acanthamoeba* species (*A. palestinensis*, *A. lugdunensis*, and *A. polyphaga*) were detected in four patients (12.9%) and flagged for clinical attention as potential pathogens given the devastating consequences of *Acanthamoeba* keratitis and the anatomical plausibility of lacrimal sac reservoir. Other parasites detected included *Balamuthia mandrillaris*, *Caenorhabditis becei*, *Leishmania tropica*, *Strongyloides venezuelensis*, and *Reticulomyxa filosa*; these were considered likely environmental contaminants or sequence artifacts due to their unprecedented occurrence in dacryocystitis, very low read counts, and lack of biological plausibility, pending orthogonal verification.

Resistance genes were detected in three patients, with the ermB gene identified in two patients and the AAC(6')-Iak gene in one patient. These genotypic findings were correlated with phenotypic resistance patterns where available and interpreted as indicators of potential antimicrobial resistance determinants regardless of organism viability.

## DISCUSSION

In this study, we utilized probe capture-based tNGS technology for the first time to comprehensively characterize the microbial landscape of lacrimal sac secretions obtained intraoperatively from patients with dacryocystitis, and compared its performance with conventional bacterial culture methods. The analysis of samples from 31 patients revealed that bacteria were the predominant microorganisms identified. The positive detection rate was 38.7% for bacterial culture and 87.1% for tNGS, indicating a significant advantage of tNGS over traditional culture techniques (*P* = 0.0002). It is important to note that while the high sensitivity of tNGS inevitably carries a risk of reduced specificity—a limitation shared by all NGS-based approaches—our use of a curated pathogen reference database containing over 26,000 clinically relevant microorganisms, combined with probe capture-based targeted enrichment, substantially reduces non-specific background and improves the reliability of pathogen identification compared to untargeted shotgun metagenomics. Beyond the expected finding of superior bacterial detection, our study revealed that the microbial landscape of the lacrimal sac extends substantially beyond bacteria to include fungi, viruses, and parasites. Notably, bacterial cultures identified *Staphylococcus epidermidis* as the most frequent bacterium (4/13), whereas tNGS revealed *Streptococcus pneumoniae* as the most common (6/31). In addition, tNGS detected *Malassezia restricta* as the most prevalent fungus (25.8%) and highlighted high detection rates of EBV (58.1%) and HHV-7 (41.9%). Moreover, parasites were detected in six patients (19.4%) through tNGS, including four cases of *Acanthamoeba* species—a finding of particular clinical concern given the devastating and treatment-refractory nature of *Acanthamoeba* keratitis. These findings suggest that the lacrimal sac harbors a complex polymicrobial ecosystem that conventional methods substantially underestimate, and raise important questions about the potential role of non-bacterial microorganisms in the pathogenesis of dacryocystitis.

Secondary dacryocystitis caused by NLDO is one of the most frequent lacrimal drainage disorders encountered in ophthalmology. Patients typically present with symptoms such as epiphora and increased ocular discharge. Acute exacerbations are often accompanied by conjunctival hyperemia and purulent discharge (12). Understanding the microbial landscape in dacryocystitis patients is crucial, as antibiotics are routinely used to manage ocular symptoms and complement surgical intervention. Accurate microbial identification helps guide antibiotic selection, reducing the risk of antimicrobial resistance and improving surgical outcomes. Previous studies have primarily used traditional bacterial culture and susceptibility testing to assess microbial distribution. Sample collection typically involves either direct conjunctival swabbing or collecting reflux secretions from the lacrimal duct by applying pressure over the lacrimal sac (13). Bacterial culture, however, shows relatively low positivity rates. For example, our previous research reported a 38.2% positivity rate using conjunctival swabs (3), while another study showed a 49% positivity rate (14). The positive rate of bacterial cultures in the current study was 38.7%, consistent with earlier findings. Due to the limitations of bacterial culture—where only a limited spectrum of microorganisms is typically isolated—its utility in guiding comprehensive antibiotic selection remains constrained.

With advancements in sequencing technology, mNGS is increasingly being applied in infectious disease diagnostics. However, untargeted mNGS suffers from high background noise from human host DNA and environmental contaminants, which can compromise specificity and complicate clinical interpretation. In contrast, tNGS employs probe capture-based enrichment targeting a predefined panel of clinically relevant pathogens, thereby reducing data complexity, lowering cost, and—critically—enhancing the signal-to-noise ratio for pathogens of interest (15). This targeted strategy is particularly advantageous in paucimicrobial specimens such as lacrimal sac secretions, where the microbial biomass is low relative to host background. In this study, tNGS demonstrated a significantly higher detection rate for bacteria, with 87.1% of samples testing positive, compared to 38.7% for traditional bacterial culture (*P* = 0.0002). While bacterial cultures identified *Staphylococcus epidermidis* as the most common bacterium, followed by *Stenotrophomonas maltophilia* and *Streptococcus pneumoniae*, tNGS detected *Streptococcus pneumoniae* as the most prevalent bacterium, followed by *Staphylococcus aureus* and *Cutibacterium acnes*. The discrepancy in the predominant organism identified by each method may reflect differences in culturability, organism viability, microbial nucleic acid abundance, sequencing reporting thresholds, and bioinformatic filtering. For example, *S. epidermidis* grows readily on standard media and may be recovered by culture even when its nucleic acid abundance is low or below the tNGS reporting threshold, whereas fastidious organisms such as S. pneumoniae and anaerobes like *C. acnes* may be missed by culture but captured by tNGS. While most previous studies have reported *Staphylococcus* species as the predominant pathogen in dacryocystitis (2), some literature also recognizes *Streptococcus* species as significant pathogens (16). These discrepancies could be attributable to variations in detection methodologies, sampling populations, geographic regions, or laboratory techniques.

Rigorous measures were implemented at both the sampling and analytical phases to minimize contamination risk. During intraoperative collection, lacrimal sac secretions were aspirated using sterile syringes before lacrimal irrigation or contact with nasal secretions; the syringe tip was maintained within the lacrimal sac opening and carefully shielded from nasal mucosa, contaminated instruments, and periocular skin. At the molecular phase, all glass beads underwent DNase/RNase-free water washing, UV irradiation (30 min), and autoclaving (121°C, 20 min) to eliminate exogenous microbial DNA. Negative extraction controls (ultrapure water processed alongside clinical samples) and no-template PCR controls were included in each tNGS batch; all controls yielded no detectable pathogen reads above the reporting threshold. Despite these precautions, we acknowledge inherent limitations in distinguishing true lacrimal sac residents from contaminants. Potential contamination sources include (i) nasal cavity microbiota introduced during endoscopic surgical access, despite visual confirmation of syringe placement; (ii) reagent-derived “kitome” contamination, a documented challenge in low-biomass microbiome studies where commercial extraction kits and PCR reagents may contain trace bacterial/fungal DNA; (iii) environmental or skin commensals (e.g., *Cutibacterium acnes* and *Staphylococcus epidermidis*) introduced during patient positioning or specimen handling; and (iv) cross-contamination between samples during multiplexed sequencing, mitigated by unique dual-index barcoding but not entirely eliminable. To aid interpretation, we applied multiple lines of evidence to distinguish plausible pathogens from contaminants: (i) relative abundance—organisms with high RPM values and dominant profiles were prioritized; (ii) clinical concordance—correlation with purulence, inflammation severity, and culture results; (iii) established pathogenicity—*Streptococcus pneumoniae*, *Staphylococcus aureus*, and *Acanthamoeba* species were weighted more heavily than common skin commensals; (iv) probe capture specificity—our targeted panel excluded common environmental sequences, reducing false positives versus untargeted metagenomics; and (v) reproducibility—detection of the same organism in technical replicates (where available) supported authenticity. Specific unexpected findings warrant cautious interpretation: the detection of *Caenorhabditis becei*, *Leishmania tropica*, *Strongyloides venezuelensis*, and *Reticulomyxa filosa*—unprecedented in dacryocystitis literature—most likely represent environmental contaminants, sequence misclassification, or reagent-derived artifacts rather than true infection. These organisms lack biological plausibility as lacrimal pathogens and were present at very low read counts. We explicitly flag these as “unverified by orthogonal methods” and recommend species-specific PCR, serology, or histopathology for confirmation before clinical action. Conversely, the high prevalence of EBV (58.1%) and HHV-7 (41.9%) may reflect nasolacrimal-nasopharyngeal anatomical continuity and viral latency/reactivation rather than active infection, though this hypothesis requires prospective validation.

A critical challenge in interpreting tNGS data from mucosal surfaces is distinguishing active infection from commensal colonization or asymptomatic carriage. We applied the following hierarchical criteria to classify detected microorganisms: (i) clinical-pathological correlation—organisms were more likely considered pathogens when associated with acute inflammatory signs (purulent discharge, lacrimal sac distension, and periocular erythema) or when they were the sole or dominant species detected; (ii) culture concordance—organisms recovered by both tNGS and conventional culture were weighted more heavily as probable pathogens, whereas tNGS-only detections, particularly at low read counts, were considered presumptive colonizers or contaminants; (iii) established pathogenicity—*Streptococcus pneumoniae*, *Staphylococcus aureus*, *Pseudomonas* species, and *Acanthamoeba* species were classified as likely pathogens based on extensive dacryocystitis literature, whereas *Cutibacterium acnes*, *Corynebacterium species*, and Malassezia species were considered probable commensals or opportunists unless present at high abundance with supporting clinical features; (iv) antibiotic response correlation—for patients with prior antibiotic exposure, organisms persisting despite therapy were flagged as potential resistant pathogens, whereas those eliminated by empirical therapy were considered likely susceptible colonizers; and (v) multi-kingdom context—bacterial-viral co-detections (e.g., *S. pneumoniae* with EBV) were interpreted as potentially synergistic pathogenic combinations, whereas isolated viral detections without inflammatory correlates were attributed to latent or subclinical carriage. We explicitly acknowledge that these criteria represent probabilistic judgments rather than definitive proof, and quantitative tNGS with absolute abundance thresholds—currently unavailable for ocular specimens—would strengthen this distinction in future studies.

We explicitly note that definitive distinction between infection and colonization in dacryocystitis requires longitudinal studies correlating microbial profiles with treatment response and clinical outcomes, as well as quantitative tNGS with validated abundance thresholds—neither of which was feasible in this cross-sectional study. Our classifications represent working hypotheses to guide clinical interpretation rather than conclusive diagnoses.

Prior antibiotic exposure is another important factor that may have influenced the microbiological findings in this study. More than 80% of patients had used topical or systemic antibiotics before specimen collection. Such exposure could suppress bacterial growth and reduce culture positivity, particularly for fastidious or low-abundance organisms. In contrast, tNGS can detect microbial nucleic acids regardless of organism viability and may therefore remain positive after antibiotic treatment. This difference may partly explain the higher detection rate of tNGS compared with conventional culture and the discordance between the two methods. Prior antibiotic exposure may also have altered the microbial composition of lacrimal sac secretions, potentially reducing susceptible bacteria while enriching resistant organisms or residual microbial DNA. Variations in clinical treatment histories, limited reliability of self-reported data, and irregular antibiotic use precluded meaningful subgroup analysis of antibiotic exposure.

Beyond species identification, tNGS offers the additional advantage of simultaneously detecting AMR genes, providing a genotypic complement to phenotypic antibiotic susceptibility testing. In this study, tNGS identified two resistance genes—ErmB and AAC(6′)-Iak—in three samples, suggesting potential resistance to macrolides in two *Streptococcus pneumoniae* samples (17) and resistance to aminoglycosides in one *Stenotrophomonas maltophilia* sample (18). Antibiotic susceptibility testing from bacterial culture revealed one case of MRSA and one case of multidrug-resistant *Streptococcus pneumoniae*. Discordance between tNGS resistance-gene detection and phenotypic AST may occur because tNGS detects resistance genes from microbial nucleic acids regardless of organism viability, gene detection does not necessarily indicate gene expression, and phenotypic AST requires viable cultured isolates. Conversely, the tNGS panel may not cover all resistance mechanisms responsible for phenotypic resistance. Therefore, resistance-gene findings should be interpreted as complementary to culture-based AST rather than as a direct substitute. The detection of AMR genes directly from clinical specimens may provide supplementary information on potential antimicrobial resistance, particularly in patients who have already received empirical antibiotics that may reduce culture positivity. Furthermore, *Stenotrophomonas maltophilia* is intrinsically resistant to multiple antibiotic classes including carbapenems, and its detection in three patients provides actionable information for antibiotic selection, as conventional empirical regimens are often ineffective against this organism. Taken together, these findings highlight that comprehensive microbiological diagnosis—integrating both genotypic resistance gene profiling and phenotypic AST—can provide clinicians with critical information to guide targeted antibiotic therapy, but should be interpreted together with culture-based susceptibility results and clinical context rather than as a standalone basis for antibiotic selection.

While most research on dacryocystitis has focused on bacterial distribution (19), other microorganisms—such as fungi, viruses, and parasites—have received considerably less attention (20). Given the nasolacrimal duct’s anatomical connection to both the ocular surface and nasal cavity (21), where viral and fungal infections are prevalent, further investigation into these microorganisms is warranted (22). For instance, Ali’s metagenomic analysis of 10 lacrimal sac samples from patients with primary acquired NLDO identified bacteria as the most common organisms, followed by viruses and archaea (23). Another study on patients with failed DCR reported BeAn 58058 virus as the most abundant virus and occasional detection of *Plasmodium* and *Toxoplasma* species (24). In the present study, tNGS—with its advantages of shorter detection time and broader detection range compared to 16S rRNA gene sequencing (25)—detected *Malassezia restricta* in eight patients (25.8%). This fungus, a recognized component of the sinus microbiome, is frequently associated with chronic rhinosinusitis, which may explain its presence in the lacrimal sac given the anatomical proximity to the paranasal sinuses (26). The high prevalence of *M. restricta* suggests that fungal co-infections or colonization may be underrecognized in dacryocystitis and could contribute to persistent inflammation in some patients, particularly those who fail to respond to antibacterial therapy alone.

Among the most striking findings of this study was the high prevalence of viral DNA in lacrimal sac secretions. EBV was detected in 18 patients (58.1%) and HHV-7 in 13 patients (41.9%). The detection of EBV at such a high frequency is particularly intriguing given the well-documented elevated prevalence of EBV in Guangdong, China, and its established causal association with nasopharyngeal carcinoma (27). Although our study cannot establish causality, the predominance of EBV—a virus known to establish lifelong latency and modulate host immune responses—raises the hypothesis that chronic viral infection or reactivation within the lacrimal sac may contribute to local inflammation, mucosal dysfunction, and predisposition to bacterial superinfection in NLDO. Similarly, HHV-7, which is known to cause various human diseases including febrile syndromes, skin lesions, neurological disorders, and post-transplant complications, may play an underrecognized role in the pathophysiology of NLDO that merits dedicated investigation (28). The co-detection of these herpesviruses in a substantial proportion of patients suggests that the lacrimal sac microbial ecosystem is intimately connected to that of the nasal cavity and nasopharynx, and viral-bacterial interactions may be important drivers of chronic lacrimal inflammation.

Equally significant is the detection of *Acanthamoeba* species in four patients (12.9%). *Acanthamoeba* keratitis is one of the most devastating corneal infections in ophthalmology, characterized by extreme pain, prolonged clinical course, and frequent resistance to standard antimicrobial therapy; delayed diagnosis is a well-established risk factor for poor visual outcomes and corneal perforation (29). The identification of *Acanthamoeba* DNA in the lacrimal sac of patients with dacryocystitis suggests—for the first time, to our knowledge—that the lacrimal sac may function as an unrecognized reservoir for these amoebae. This reservoir hypothesis carries profound clinical implications: amoebae harbored in the lacrimal sac could periodically reflux onto the ocular surface, potentially initiating or perpetuating corneal infection in susceptible individuals, and may explain some cases of recurrent or refractory *Acanthamoeba* keratitis in which the source of infection remains elusive (30). Furthermore, the detection of multiple *Acanthamoeba* species (*A. rhysodes*, *A. palestinensis, A. lugdunensis*, and *A. polyphaga)* in different patients indicates that this phenomenon is not limited to a single species. These observations fundamentally challenge the traditional view that dacryocystitis is exclusively a bacterial disease and open new avenues for understanding the etiology, pathogenesis, and management of both lacrimal and corneal infections.

Collectively, the findings of this study support a broader, hypothesis-generating view of the microbiology of dacryocystitis. The traditional bacteriology-centered framework—in which dacryocystitis is viewed primarily as a consequence of nasolacrimal duct obstruction with secondary bacterial overgrowth—may be insufficient to capture the full complexity of the disease. Our data suggest that dacryocystitis may involve a more complex polymicrobial ecosystem, in which bacteria, fungi, viruses, and parasites may interact within the lacrimal sac, potentially contributing to chronic inflammation and tissue damage. Within this framework, tNGS serves not merely as a more sensitive replacement for culture, but as a discovery tool that can reveal previously unsuspected microbial players and generate testable hypotheses about disease mechanisms. Several priority areas for future research emerge from this work: (i) longitudinal studies to determine whether the microbial profiles identified by tNGS predict clinical outcomes, treatment response, or postoperative complications; (ii) quantitative tNGS with standardized abundance and pathogenicity thresholds to differentiate active infection from colonization; (iii) investigation of viral-bacterial and amoeba-bacterial interactions within the lacrimal sac microenvironment; (iv) multi-center studies to assess geographic and population-level variability in lacrimal sac microbial communities; and (v) clinical trials to evaluate whether tNGS-guided antimicrobial therapy improves patient outcomes compared to empirical treatment. Addressing these questions will be essential to translate the comprehensive microbial profiling enabled by tNGS into tangible clinical benefits for patients with dacryocystitis.

The viral, parasitic, and other unexpected taxa detected by tNGS should be interpreted as nucleic-acid findings rather than definitive evidence of active infection or pathogenicity. A critical consideration in interpreting these tNGS findings is the differentiation between potential causative pathogens and organisms that may represent commensal flora, environmental contaminants, or transient colonizers. Many of the bacteria detected by tNGS—including *Cutibacterium acnes*, *Staphylococcus epidermidis*, and *Corynebacterium species*—are well-documented components of the normal ocular surface and periocular skin microbiome. Their detection in lacrimal sac samples does not necessarily indicate a pathogenic role. Several lines of evidence can help distinguish pathogens from commensals in tNGS data: (i) relative abundance—pathogens typically predominate in clinical infection, whereas commensals are present at lower, consistent levels; (ii) the presence of a single dominant organism rather than a mixed flora profile may suggest a pathogenic role; (iii) correlation with clinical severity and purulence; (iv) concordance with culture results when the organism is culturable; and (v) comparison with established literature on dacryocystitis-associated pathogens. In our study, organisms such as *Streptococcus pneumoniae* and *Staphylococcus aureus*—which were detected at high relative abundance and are established pathogens in dacryocystitis—are more likely to represent true infections, whereas organisms such as *C. acnes* and *S. epidermidis* may represent commensal flora or secondary colonizers. It is important to emphasize that the probe capture-based targeted approach employed in this study, which uses a curated database of clinically relevant microorganisms, inherently reduces the detection of spurious environmental sequences compared to untargeted shotgun metagenomics, thereby strengthening the reliability of positive findings. Nonetheless, future studies incorporating quantitative tNGS with standardized abundance thresholds, paired sampling from unaffected contralateral lacrimal sacs or ocular surfaces, and clinical outcome correlation would greatly facilitate this distinction.

Regarding the detection of parasites, several points warrant careful consideration. The identification of *Caenorhabditis becei*, *Leishmania tropica*, *Strongyloides venezuelensis*, and *Reticulomyxa filosa* in lacrimal sac samples is, to our knowledge, unprecedented in the dacryocystitis literature. *Caenorhabditis* species are free-living soil nematodes not typically associated with human infection; their detection may reflect environmental contamination, sequence misclassification, or laboratory reagent-derived sequences rather than true lacrimal sac infection. *Leishmania tropica* is a known cause of cutaneous leishmaniasis, and ocular involvement—though rare—has been documented in the form of eyelid or conjunctival lesions; however, its presence in the lacrimal sac has not been previously reported, and confirmation through parasite-specific PCR or serological testing would be required to establish clinical significance. *Strongyloides* species are intestinal nematodes, and although ocular strongyloidiasis has been rarely reported in the context of disseminated infection in immunocompromised patients, its detection by tNGS alone should be interpreted with caution. *Reticulomyxa filosa* is also unexpected in this clinical context and may represent an environmental or database-related artifact. More broadly, these findings highlight a fundamental limitation of highly sensitive NGS-based methods: they detect microbial nucleic acids without distinguishing viable from non-viable organisms, pathogenic from non-pathogenic species, active infection from transient passage or contamination, or true taxonomic assignment from sequence misclassification. We therefore recommend that unusual or unexpected tNGS findings be confirmed by orthogonal methods—such as species-specific PCR, serology, or histopathological examination—before guiding clinical treatment decisions.

This study has several limitations that should be acknowledged. First, the sample size was relatively small (*n* = 31), and the cohort included patients with various forms of dacryocystitis (acute, chronic, and congenital), which may have introduced heterogeneity in the observed microbial distribution and may limit the generalizability of our findings. In addition, this pilot study was not powered for formal hypothesis testing; the modest sample size (*n* = 31) yields wide confidence intervals for prevalence estimates (e.g., the 95% CI for tNGS bacterial positivity spans 70.2%–95.1%). Effect sizes should be interpreted with caution, and the reported *P*-values are descriptive rather than confirmatory. No correction for multiple comparisons was applied, increasing the risk of type I error when interpreting the broad range of microbial taxa detected. These findings require validation in larger, multi-center cohorts with prespecified primary endpoints. Second, the study was conducted at a single tertiary ophthalmic center in southern China, and the microbial profiles observed—particularly the high prevalence of EBV—may reflect regional epidemiological characteristics that are not representative of other populations. Third, this study did not include healthy lacrimal sac or conjunctival control samples, which limits our ability to distinguish disease-associated microorganisms from normal ocular microbiota, transient colonizers, or background contaminants. In addition, the tNGS method used in this study provides qualitative or semi-quantitative microbial detection without absolute quantification of microbial load; the absence of established abundance thresholds for defining clinically significant infection means that the distinction between true pathogens, commensals, and contaminants remains challenging and requires clinical correlation. Fourth, a substantial proportion of patients (80.6%) had received topical and/or systemic antibiotics prior to sample collection, which may have altered the microbial profile and reduced the sensitivity of bacterial culture. Fifth, lacrimal sac secretions were collected intraoperatively after incision of the lacrimal sac during En-DCR, which carries a potential risk of contamination from the nasal cavity despite precautions taken during sampling. Ideally, preoperative percutaneous aspiration of lacrimal sac contents would provide a more representative microbiological sample; however, this approach was not feasible in the present study due to ethical and practical constraints, as all patients required surgical intervention. Sixth, the cross-sectional design precludes assessment of temporal changes in the lacrimal sac microbiome and the effect of surgical intervention on microbial clearance. Finally, the absence of standardized interpretive criteria for tNGS in ocular specimens means that positive results—particularly for unusual or unexpected organisms—should be validated by orthogonal methods before guiding clinical management. Large-scale, multi-center, prospective studies incorporating quantitative tNGS, paired sampling from multiple anatomical sites, and clinical outcome data are needed to establish definitive criteria for interpreting tNGS results in dacryocystitis and other ocular infections.

### Conclusion

This study establishes probe capture-based tNGS as a valuable complementary tool to conventional culture for microbial detection in dacryocystitis, achieving higher detection rates (87.1% versus 38.7%) and broader coverage across bacteria, fungi, viruses, and parasites while simultaneously identifying resistance genes. These findings illuminate the lacrimal sac as a complex polymicrobial ecosystem, challenging the traditional bacteriology-centered paradigm and raising novel hypotheses—including chronic viral contribution to nasolacrimal duct obstruction pathogenesis and the lacrimal sac as a potential reservoir for *Acanthamoeba* and other ocular surface pathogens. While prospective validation is needed to correlate molecular profiles with clinical outcomes, this work lays the foundation for a microbiome-informed approach to precision diagnosis and targeted therapy of dacryocystitis.

## Data Availability

The sequencing data generated and analyzed during the current study have been deposited in the NCBI Sequence Read Archive (SRA) under the BioProject accession number PRJNA1274608. The data set is publicly accessible at: https://www.ncbi.nlm.nih.gov/sra/PRJNA1274608.

## SUPPLEMENTAL MATERIAL

Table S1 spectrum.02132-26-s0001.docx Clinical and microbiological characteristics of 31 patients with dacryocystitis.

ASM does not own the copyrights to Supplemental Material that may be linked to, or accessed through, an article. The authors have granted ASM a non-exclusive, world-wide license to publish the Supplemental Material files. Please contact the corresponding author directly for reuse.
