# Supplementary material for: Probe capture-based tNGS for microbial detection of dacryocystitis
Source: Microbiol Spectr. 2026 Sep 21;14(11):e02132-26. doi: 10.1128/spectrum.02132-26 (PMC13729140; doi:10.1128/spectrum.02132-26)
Supplement: Supplementary file 1 [file spectrum.02132-26-s0001.docx]

**Table S1. Clinical and microbiological characteristics of 31 patients with dacryocystitis**

| Characteristic | n (%) or median (IQR) | 95% CI or range |  |
| --- | --- | --- | --- |
| Demographics |  |  |  |
| Age | years | 50.5 (38.0–62.0) | 18–78 |
| Female sex | 26 (83.9%) | 66.3–94.5% |  |
| Clinical features |  |  |  |
| Disease duration | months | 36.0 (12.0–96.0) | 0.5–360 |
| Right eye involvement | 11 (35.5%) | 19.2–54.6% |  |
| Left eye involvement | 17 (54.8%) | 36.0–72.7% |  |
| Bilateral involvement | 3 (9.7%) | 2.1–25.4% |  |
| Tearing | 25 (80.6%) | 62.5–92.5% |  |
| Increased ocular discharge | 29 (93.5%) | 78.6–99.2% |  |
| Purulent discharge | 28 (90.3%) | 73.9–97.9% |  |
| Prior treatment |  |  |  |
| Any prior antibiotic use | 25 (80.6%) | 62.5–92.5% |  |
| Oral antibiotics | 6 (19.4%) | 7.5–37.5% |  |
| Intravenous antibiotics | 1 (3.2%) | 0.1–16.7% |  |
| Antibiotic eye drops | 18 (58.1%) | 38.9–75.5% |  |
| Prior lacrimal surgery | 5 (16.1%) | 5.5–34.0% |  |
| Surgical intervention |  |  |  |
| En-DCR alone | 5 (16.1%) | 5.5–34.0% |  |
| En-DCR with silicone intubation | 26 (83.9%) | 66.3–94.5% |  |
| Microbiological detection |  |  |  |
| Bacterial culture positive | 12 (38.7%) | 22.4–57.5% |  |
| tNGS bacterial positive | 27 (87.1%) | 70.2–95.1% |  |
| tNGS fungal positive | 20 (64.5%) | 45.4–80.8% |  |
| tNGS viral positive | 22 (71.0%) | 52.0–85.8% |  |
| tNGS parasite positive | 6 (19.4%) | 7.5–37.5% |  |
| tNGS resistance gene positive | 3 (9.7%) | 2.1–25.4% |  |

*Note: Percentages are calculated with n=31 as denominator. For the three patients with bilateral dacryocystitis, the right eye was selected for sampling and analysis. Confidence intervals for proportions are calculated using the Clopper–Pearson exact method.*
